# Supplementary material for: Modeling glioblastoma heterogeneity as a dynamic network of cell states
Source: Mol Syst Biol. 2021 Sep 16;17(9):e10105. doi: 10.15252/msb.202010105 (PMC8444284; doi:10.15252/msb.202010105)
Supplement: Supplementary file 5 — Source Data for Figure 3 [file MSB-17-e10105-s001.zip › Figure3A_sourcedata/GSEA_3065/nosigdb_state1.GseaPreranked.1622718409535/NB_KI_CLUSTER1.html]

Details for gene set NB\_KI\_CLUSTER1[GSEA]

|  || Dataset | state1 |
| Phenotype | NoPhenotypeAvailable |
| Upregulated in class | na\_pos |
| GeneSet | NB\_KI\_CLUSTER1 |
| Enrichment Score (ES) | 0.859907 |
| Normalized Enrichment Score (NES) | 2.5576336 |
| Nominal p-value | 0.0 |
| FDR q-value | 0.0 |
| FWER p-Value | 0.0 |
Table: GSEA Results Summary

  

Fig 1: Enrichment plot: NB\_KI\_CLUSTER1      
 Profile of the Running ES Score & Positions of GeneSet Members on the Rank Ordered List

  

| PROBE | GENE SYMBOL | GENE\_TITLE | RANK IN GENE LIST | RANK METRIC SCORE | RUNNING ES | CORE ENRICHMENT || 1 | RPS12 |  |  | 73 | 0.411 | 0.0491 | Yes |
| 2 | RPS2 |  |  | 124 | 0.342 | 0.0910 | Yes |
| 3 | RPL28 |  |  | 145 | 0.326 | 0.1338 | Yes |
| 4 | RPS18 |  |  | 148 | 0.325 | 0.1783 | Yes |
| 5 | RPS6 |  |  | 151 | 0.321 | 0.2222 | Yes |
| 6 | RPL41 |  |  | 191 | 0.301 | 0.2597 | Yes |
| 7 | RPL18A |  |  | 198 | 0.296 | 0.2998 | Yes |
| 8 | RPS8 |  |  | 210 | 0.291 | 0.3387 | Yes |
| 9 | TPT1 |  |  | 220 | 0.287 | 0.3773 | Yes |
| 10 | RPS3 |  |  | 222 | 0.287 | 0.4167 | Yes |
| 11 | RPL32 |  |  | 263 | 0.274 | 0.4503 | Yes |
| 12 | RPS15A |  |  | 323 | 0.252 | 0.4790 | Yes |
| 13 | RPS7 |  |  | 331 | 0.250 | 0.5127 | Yes |
| 14 | RPLP1 |  |  | 341 | 0.244 | 0.5453 | Yes |
| 15 | RPS27A |  |  | 354 | 0.239 | 0.5769 | Yes |
| 16 | RPLP2 |  |  | 356 | 0.239 | 0.6097 | Yes |
| 17 | RPS19 |  |  | 416 | 0.223 | 0.6343 | Yes |
| 18 | RPS25 |  |  | 431 | 0.221 | 0.6633 | Yes |
| 19 | RPL13 |  |  | 434 | 0.220 | 0.6934 | Yes |
| 20 | RPL27A |  |  | 455 | 0.214 | 0.7209 | Yes |
| 21 | RPS3A |  |  | 476 | 0.211 | 0.7478 | Yes |
| 22 | RPS4X |  |  | 549 | 0.196 | 0.7674 | Yes |
| 23 | RPL23A |  |  | 612 | 0.184 | 0.7864 | Yes |
| 24 | RPL21 |  |  | 660 | 0.177 | 0.8059 | Yes |
| 25 | RPL10 |  |  | 664 | 0.176 | 0.8299 | Yes |
| 26 | RPL39 |  |  | 718 | 0.170 | 0.8478 | Yes |
| 27 | RPL3 |  |  | 811 | 0.156 | 0.8599 | Yes |
| 28 | RPL34 |  |  | 1023 | 0.131 | 0.8565 | No |
| 29 | RPS27 |  |  | 1369 | 0.098 | 0.8350 | No |
| 30 | RPL13A |  |  | 1559 | 0.085 | 0.8275 | No |
| 31 | RPS29 |  |  | 1577 | 0.084 | 0.8374 | No |
| 32 | EEF1A1 |  |  | 6584 | -0.040 | 0.3346 | No |
Table: GSEA details [plain text format]

  

Fig 2: NB\_KI\_CLUSTER1: Random ES distribution      
 Gene set null distribution of ES for **NB\_KI\_CLUSTER1**
